# First Report of a Novel Goose Adenovirus Outbreak in Lion Head Gooses in China

**DOI:** 10.1155/2024/3980468

**Published:** 2024-02-08

**Authors:** Rongchang Liu, Minhua Sun, Qin Lan, Jiaxue Zhang, Qizhang Liang, Guanghua Fu, Ming Liao, Yu Huang

**Affiliations:** ^1^Fujian Provincial Key Laboratory for Avian Diseases Control and Prevention, Institute of Animal Husbandry and Veterinary Medicine, Fujian Academy of Agricultural Sciences, Fuzhou, China; ^2^National and Regional Joint Engineering Laboratory for Medicament of Zoonosis Prevention and Control, Guangdong Provincial Key Laboratory of Zoonosis Prevention and Control, College of Veterinary Medicine, South China Agricultural University, Guangzhou, China; ^3^Institute of Animal Health, Guangdong Academy of Agricultural Sciences, Key Laboratory for Prevention and Control of Avian Influenza and Other Major Poultry Diseases, Ministry of Agriculture and Rural Affairs, Guangzhou, China; ^4^Key Laboratory of Livestock Disease Prevention and Treatment of Guangdong Province, Zhongkai University of Agriculture and Engineering, Guangzhou, China

## Abstract

In April 2022, a novel *Goose adenovirus* (GoAdV) isolated from diseased Lion head gooses exhibiting swelling and hemorrhage of liver and kidney, accumulation of fluid in pericardial, in Fujian province of China. The GoAdV was propagated in goose embryo fibroblasts (GEFs), the morphological properties of the virions were studied by electron microscopy, and the full genome sequence was determined and analyzed. The results revealed that the infected cells became round and clustered like grapes, virions accumulated and were arranged in crystal lattice formations in the nucleus of GEFs with a diameter of ∼80 nm. The new isolate (named CH-FJZZ-202201) has a viral genome size of 43,480 bp and shared 96.69% sequence identity with GoAdV-4 (P29), representing the species *Goose aviadenovirus A*. Phylogenetic analysis showed that CH-FJZZ-202201 was in the same genetic evolutionary branch with the viruses of *Aviadenovirus* and was the closest relative to GoAdV-4 P29/Hungary. This is the first report of the GoAdV-4 outside of Hungary, indicating the reemergence of new AdV strains in China.

## 1. Introduction

Adenoviruses are nonenveloped viruses with icosahedral virions containing linear dsDNA genomes that infect members of every class of vertebrates from fish to mammals. The family Adenoviridae contains the genera *Atadenovirus*, *Aviadenovirus*, *Ichtadenovirus*, *Mastadenovirus*, *Siadenovirus*, and *Testadenovirus* ([1]; http://ictv.global/report/adenoviridae). The *Atadenovirus* genus infects reptiles, birds, ruminants, and marsupials; *Aviadenovirus* genus infects only birds; *Ichtadenovirus* has been reported in fish; *Mastadenovirus* genus infects various mammalian species, such as monkeys, sheep, and cattle; *Siadenovirus* has been reported in birds, frogs, and tortoises; *Testadenovirus* has been reported in tortoise only ([2]; http://ictv.global/report/adenoviridae).

The genus *Aviadenovirus* consists of 16 officially accepted species, grouping of types into species is as follows: *Aviadenovirus leucophthalmi* (Southern *Psittacara leucophthalmus aviadenovirus* and white-eyed parakeet adenovirus 2), *Falcon adenovirus A* (FaAdV-1), *Fowl adenovirus A* (FAdV-1), *Fowl adenovirus B* (FAdV-5), *Fowl adenovirus C* (FAdV-4 and FAdV-10), *Fowl adenovirus D* (FAdV-2, FAdV-3, FAdV-9, and FAdV-11), *Fowl adenovirus E* (FAdV-6, FAdV-7, FAdV-8a, and FAdV-8b), *Goose adenovirus A* (GoAdV-4), *Pigeon adenovirus A* (PiAdV-1), *Pigeon adenovirus B* (PiAdV-2), *Psittacine adenovirus B* (PsAdV-4), *Psittacine adenovirus C* (PsAdV-1), *Turkey adenovirus B* (TAdV-1), *Turkey adenovirus C* (TAdV-4), and *Turkey adenovirus D* (TAdV-5; [2]; http://ictv.global/report/adenoviridae).

Several adenoviruses have been identified or detected in waterfowl (including ducks and geese). DAdV-1 (egg drop syndrome virus, EDSV) belongs to the genus *Atadenovirus* and common in geese and ducks [3, 4]. DAdV-2 was first isolated in 1,977 from diseased Moscow ducks in France, and the virus caused a large outbreak in the local ducks [5, 6]. DAdV-3 was first isolated in Chinese Muscovy ducks in 2016 and was classified as the *Aviadenovirus* DAdV-B species along with DAdV-2 [2, 7]. GoAdV-4 strain P29 was isolated from died goslings in Hungary during the 1970s, and its complete genome sequence were determined in 2012 [8]. GoAdV-5 strains have been found that caused hepatitis and hydropericardium syndrome (HPS) in young goslings in Hungary [9]. A dsDNA virus belonging to the family Adenoviridae and ∼75%–86% identical to GoAdV-4 (JF510462) from Hungary and DAdV-2 (KR135164) from China was found in the MAD fecal sample, during metagenomics studies on fecal samples from Australian wild birds [10]. In addition, FAdV-4 strains isolated from geese with inclusion body hepatitis (IBH) and HPS have been reported in China [11]. An epidemiological survey conducted recently on apparently healthy birds in China also showed that FAdV-4 were detected in duck and goose samples, while FAdV-8B was detected in duck samples [12].

In this study, we first isolated a novel GoAdV from the diseased Lion head gooses in Fujian province of China, in 2022. To deepen our understanding of the sequence characteristics of this novel virus, the complete genome sequence of GoAdV-4 (CH-FJZZ-202201) was obtained using high-throughput sequencing technology. Additionally, the culture and morphological characteristics of this emerging GoAdV-4 were further researched. These findings also verified the need to study and monitor GoAdV-4, which circulates in China.

## 2. Materials and Methods

### 2.1. Sample Collection and Bacterial Culture

The heart, liver, spleen, and kidney tissues from the diseased Lion head gooses in commercial flocks in Fujian province of China were collected. The collected samples were used for bacterial culture and virus detection and isolation. For bacteriological diagnosis, heart, liver, spleen, and kidney samples from dead goslings were first inoculated onto tryptic soy agar plates (BD Science, MD, USA) containing 2% fetal calf serum and incubated at 37°C under an atmosphere with 5% CO_2_ for 48 hr. Then, the samples were mixed and grinded in phosphate-buffered saline (PBS) to make 10% (*wt./vol*.) suspension. After freezing and thawing three times, the suspensions were clarified by centrifugation at 12,000x *g* for 10 min at 4°C, and then orderly filtered through 0.22 *μ*m filters (Millipore, Bedford, MA, USA). The filtered suspensions were stored at −80°C until use.

### 2.2. Virus Detection and Isolation

Total DNA and RNA were separately extracted from the homogenized tissues using a EasyPure® Viral DNA/RNA Kit according to the manufacturer's instructions (TransGen Biotech Co., Ltd., Beijing, China). DNA and RNA were subjected to PCR or RT-PCR for the detection of potential pathogens, such as avian astrovirus (AvAstV) [13], goose parvovirus (GPV) [14], reovirus (REOV) [15], goose hemorrhagic polyomavirus (GHPV) [16], tembusu virus (TMUV) [17], fowl adenovirus (FAdV) [18], duck adenovirus (DAdV) [19], and GoAdV, respectively (Table 1).

Goose embryo fibroblasts (GEFs) were prepared from 9-day-old Lion head goose embryos purchased from Baisha Livestock and Animal Original Seed Research Institute, Shantou city, Guangdong province. The isolated virus sample, which was designated CH-FJZZ-202201, was cultured in GEFs. Briefly, GEFs were cultured in 100 mm tissue culture dishes in DMEM for 24 hr. Then, the culture medium was removed, and CH-FJZZ-202201 virus was added to the medium. The virus was harvested when a 75% CPE was observed. After the cultures were blind passaged four times, viral titers were calculated using the 50% tissue culture infective dose (TCID_50_) according to the Reed and Muench method.

### 2.3. Virus Purification and Electron Microscopy

Two hundred milliliters of virus propagated in GEFs was obtained, centrifuged at 10,000x *g* for 10 min to pellet large cellular debris, and then centrifuged again at 40,000x *g* for 3 hr at 4°C to further remove debris. The purified virus was then placed on a copper grid, negatively stained with 3% phosphotungstic acid, and observed and photographed with a transmission electronic microscope (TEM, H-7500, Hitachi, Japan).

### 2.4. Genome Sequencing and Phylogenetic Analysis

An lllumina system (HiSeq 2000, BGI, Hong Kong) was used to obtain the whole complete genome of CH-FJZZ-202201. Paired-end libraries were generated, and multiple virus samples were sequenced in a single lane. The obtained reads were used to assemble the virus genome against the available GoAdV-4 nucleotide sequences of strain P29 (JF510462.1). Based on the nucleotide sequences of strains P29, a set of primers were designed to amplify and proofread the complete genome sequence of CH-FJZZ-202201 (Table 2). PCR products with expected lengths were sequenced directly or cloned into the pEASY-T1 cloning vector (TransGen, Beijing, China) according to the manufacturer's instructions and sequenced at Sangon Biotech Co., Ltd. (Shanghai, China). The nucleotide and amino acid sequences were assembled using DNASTAR (version 7; Madison, WI, USA), multiple-sequence alignment was performed with the Clustal W (BioEdit version 7) program, and online alignment in NCBI-BLAST software (https://blast.ncbi.nlm.nih.gov/Blast.cgi). A neighbor-joining (NJ) tree based on the full-length nucleic acid and hexon amino acid sequences were constructed using the MEGA5.1 program. The robustness of the NJ tree was evaluated with a bootstrap analysis of 1,000 replicates.

## 3. Results

### 3.1. Case History, Microbiological Examination, and Virus Isolation of the Field Samples

**Figure 1:**
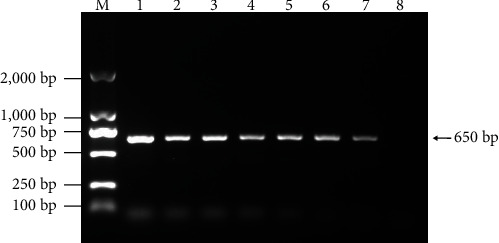
PCR assay of the infected tissues with specific primers for GoAdV. M, DNA marker DL 2000; 1–7, clinical samples; 8, negative control.

**Figure 2:**
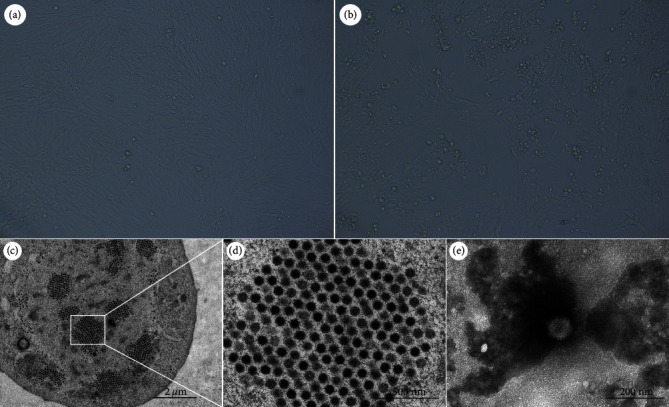
Isolation and electron micrograph of GoAdV strain CH-FJZZ-202201. (a and b) CH-FJZZ-202201 isolate-infected (a) or mock-infected-GEFs (b) were observed under a microscope. (c and d) Electron micrographs of GoAdV on a CH-FJZZ-202201-infected GEFs at 2 dpi. (e) Small icosahedra and nonenveloped particles (80 nm in diameter) were observed.

In April 2022, a 40-day-old Lion head gooses occurred in a commercial flocks in Fujian province of China, and its morbidity and mortality rate were about 20% and 80%, respectively. The diseased gooses presented mental depression, decreased food intake, emaciated back, head and neck tremor, drooping wings, and creeping on the ground within 7–14 days postinfection (dpi). At necropsy, swelling and hemorrhage appeared in the liver and kidney, sporadically appeared accumulation of clear fluid in the dilated pericardial sac. Nine tissue samples were collected in the same month, virulent bacteria were not isolated, and the samples tested negative for AvAstV, GPV, REOV, GHPV, TMUV, FAdV, and DAdV, but only positive for GoAdV as determined by PCR assays (Figure 1). The virus was isolated in GEFs, and the cells were harvested at 4 dpi. After three generations of blind transmission, cells began to show significant CPE in the fourth generation. The titer of the virus was determined as 10^−5.5^ TCID_50_. Compared to the nuclei of normal GEFs (Figure 2(a)), the nuclei of GoAdV infected cells became round and clustered like grapes (Figure 2(b)). The isolate was designated as CH-FJZZ-202201.

### 3.2. Electron Microscopy

The virus was purified using the method of limiting dilution assay and density-gradient ultracentrifugation. Electron microscopic observation showed that virions accumulated and were arranged in crystal lattice formations in the nucleus of GEFs (Figures 2(c) and 2(d)), small icosahedra and nonenveloped virus particles with a diameter of ∼80 nm present in the purified samples (Figure 2(e)).

### 3.3. Genome Sequence of Goose/CH-FJZZ-202201

**Figure 3:**
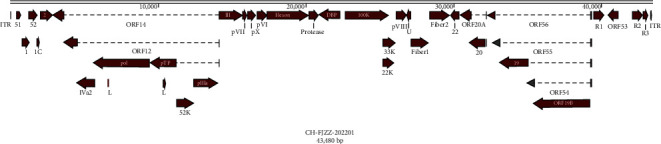
Predicted genome organization of GoAdV strain CH-FJZZ-202201.

The genome of goose/CH-FJZZ-202201 was 43,480 nucleotides (nts) in length, with a G + C content of 44.46%, and the predicted open reading frames (ORFs) were similar to GoAdV-4 strain P29 (Figure 3; Table 3). The complete nucleotide sequences were deposited to the GenBank with accession number OR842729.

### 3.4. Phylogenetic Analysis of Goose/CH-FJZZ-202201

**Figure 4:**
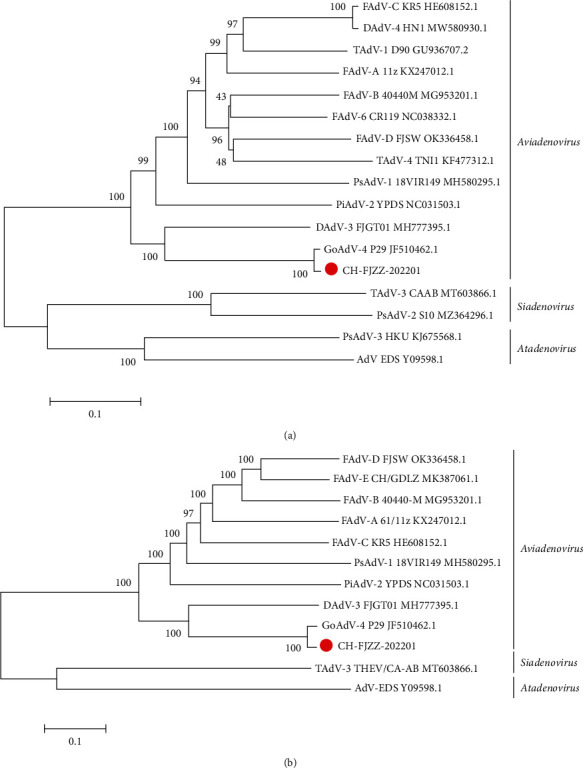
Phylogenetic analysis of strain CH-FJZZ-202201. The phylogenetic trees were constructed by the maximum likelihood method in MEGA 5.2. Bootstrap majority consensus values based on 1,000 replicates are indicated at each branch point as a percentage. (a) Phylogenetic tree of the nucleotide sequences of hexon. (b) Phylogenetic tree of the nucleotide sequences of whole genome.

The adenovirus reference genomes were downloaded from the GenBank database. The phylogenetic analysis of the hexon gene and complete genome showed that CH-FJZZ-202201 was in the same genetic evolutionary branch with the viruses of *Aviadenovirus*, such as GoAdV-4, DAdV-3, and FADV-A∼E, and were closest relative to GoAdV-4 P29/Hungary (JF510462.1; Figures 4(a) and 4(b)). Sequence homology analysis showed that the CH-FJZZ-202201 shared 99.25%, 81.91%, 72.3%, 72.26%, and 71.76% identity of its hexon amino acid sequence with GoAdV-4 (P29), DAdV-3 FJGT01 (MH777395.1), PiAdV-2 (NC031503.1), PsAdV-1 (MH580295.1), and FAdV-4 (MG856954.1), respectively. In addition, the whole genome of CH-FJZZ-202201 showed 96.69% identity to GoAdV-4 (P29) and 75.9% identity to DAdV-3 (FJGT01; MH777395.1).

## 4. Discussion

Outbreaks of AdVs have been frequently reported worldwide, leading to huge economic losses to the poultry industry [20]. In recent years, more and more AdVs have been isolated or detected from dead, sick, or apparently healthy birds [12, 20–23]. Since 2015, new AdVs subtypes have emerged in the our poultry industry, such as serotype FAdV-2 [24], FAdV-4 [25], FAdV-7 [26], FAdV-8a [27], FAdV-8b [24], FAdV-11 [26], and DAdV-3 [7]. In this study, a novel goose-origin adenovirus, provisionally designated as GoAdV-4 strain CH-FJZZ-202201, was successfully isolated from diseased Lion head gooses in China. Moreover, the cultivate characteristics, morphology, and whole genome characterization were deeply analysed.

Avian embryo fibroblasts or chicken hepatoma cell lines (LMH) were commonly used to isolate AdVs [7, 28, 29]. We attempted to isolate virus from GoAdV-positive samples using GEFs cells. Clinical samples after fourth blind passages in GEFs cells, the infected cells became nuclei, round and grapes-like clustered, and the viral titer reached 10^−5.5^ TCID_50_/mL, showing that CH-FJZZ-202201 strain was successfully obtained by separation. AdVs morphology in many species has been reported, such as chicken, duck, and pigeon source [7, 29–32]. Nevertheless, there was no report on the morphology of *Goose adenovirus A*. Our results showed that GoAdV-4 strain CH-FJZZ-202201 virions accumulated and were arranged in crystal lattice formations in the nucleus of GEFs, a small icosahedra and nonenveloped virus particles with a diameter of ∼80 nm were observed under electron microscopy, which was consistent with the general characteristics of the AdVs [2]. Our results further enrich the morphology of AdVs of different species.

AdVs was a double-stranded DNA virus, with a genome of 38–46 kb in size and the G + C content varies between 34.16% and 66.92% [2, 33, 34]. The complete DNA sequence of GoAdV strain CH-FJZZ-202201 was reported here, the sequence indicated a viral genome size of 43,480 bp, with a G + C content of 44.46%. The phylogenetic analysis of the hexon gene and complete genome showed that CH-FJZZ-202201 was in the same genetic evolutionary branch with the viruses of *Aviadenovirus* and was the closest relative to GoAdV-4 P29/Hungary (Figures 4(a) and 4(b)). According to the hexon amino acid sequence homology analysis, the CH-FJZZ-202201 was the most similar to the GoAdV-4 (P29) isolate (99.25%), followed by DAdV-3 FJGT01 (MH777395.1; 81.91%). Moreover, we compared the whole genome homology between the CH-FJZZ-202201 and other AdVs, and our result showed that CH-FJZZ-202201 was also the most similar to the GoAdV-4 (P29) isolate (96.69%), followed by DAdV-3 (FJGT01; 75.9%). These findings suggested that the CH-FJZZ-202201 might be classified into the GoAdV-4 of *Goose adenovirus A* species. This is the first report of the GoAdV-4 strains outside of Hungary, and our study has enriched the information on the GoAdV-4 virus [8].

AdVs infections are associated with a range of avian infectious diseases, normal with IBH and HPS as the main pathological features [2]. *Goose adenovirus* has been reported to have a role in IBH and HPS of goslings [9]. In the case of this study, the necropsy of the diseased 40-day-old Lion head gooses showed swelling, and hemorrhage appeared in the liver and kidney, sporadically appeared accumulation of clear fluid in the dilated pericardial sac, which is consistent with the typical pathological features of AdVs infections, HPS. Nevertheless, as the above case has not yet been reproduced by the experimental infection of goslings with those isolates, the pathogenicity of the GoAdV need to further confirmed.

In summary, a GoAdV-4 strain CH-FJZZ-202201 was successfully isolated from commercial Lion head gooses in Fujian province, China. The first complete genome of GoAdV-4 in China was determined and characterized, which not only increased the knowledge of the molecular characteristics but also enriched the understanding of GoAdV-4 diversity. We are puzzled by how this GoAdV-4 strain was introduced into goose herds and how long they had been circulating in China. We are concerned that this virus may pose a threat to the poultry industry in the future. Therefore, further studies of rapid diagnosis technology, pathogenic mechanism, and vaccine development of GoAdV-4 are needed to obtain more insights for the prevention and control of this disease.

## Figures and Tables

**Table 1:** Primers were used for detection in this study.

Primer name	Sequence (5′–3′)	Target	Reference
AvAstV-F1	GAYTGGACNMGNTAYGAYGGNACNATNCC	ORF1b gene of AvAstV (434 bp)	[13]
AvAstV-R1	YTTNACCCACATNCCRAA	—	—
GPV-F	AGACTTATCAACAACCATCA(T/C) T	VP1 gene of GPV (779 bp)	[14]
GPV-F	TCACTTATTCCTGCTGTAG	—	—
REOV-F	GGTGCGACTGCTGTATTTGGTAAC	S1 gene of REOV (513 bp)	[15]
REOV-R	AATGGAACGATAGCGTGTGGG	—	—
GHPV-F	GAGGTTGTTGGAGTGACCACAATG	VP1 gene of GHPV (144 bp)	[16]
GHPV-R	ACAACCCTGCAATTCCAAGGGTTC	—	—
TMUV-F	GCCACGGAATTAGCGGTTGT	E gene of TMUV (401 bp)	[17]
TMUV-R	TAATCCTCCATCTCAGCGGTGTAG	—	—
FAdV-1F	CAARTTCAGRCAGACGGT	Hexon gene of FAdV (897 bp)	[18]
FAdV-1R	TAGTGATGMCGSGACATCAT	—	—
FAdV-2F	SKCSACYTAYTTCGACAT	Hexon gene of FAdV (580 bp)	[18]
FAdV-2R	TTRTCWCKRAADCCGATGTA	—	—
DAdV-F	CAATTTTAAACGGTTTGTAGGTTC	Hexon gene of DAdV (1,102 bp)	[19]
DAdV-R	CATACTGGTCTACTGCCTGAT	—	—
GoAdV-F	TGTCTTTAGTAATGATGGGTAT	Hexon gene of GoAdV (580 bp)	Designed by this study
GoAdV-R	CCTCATTAAACACTACGGAT	—	—

**Table 2:** Primers used to amplify the complete genomic sequence of GoAdV-4 strain CH-FJZZ-202201.

Primer	Location (bp)	Upstream primer (5′–3′)	Downstream primer (5′–3′)	Length (bp)
1	1–1403	CATCATCATATATAAAATAACCAC	TGTATAAGTAATTTACATCATGAG	1,403
2	1278–2751	AGGATGAACGATAGGTTAGTAA	TAGATACACAGGGAGGTAGT	1,474
3	2655–4071	CATTCCTCAGAGTGTGTTTC	ACCGCCGACATTAATAATATG	1,417
4	3954–5392	TGGTTTACTACAGGGTTAGAA	CCGTCATTGGAGTCATTTAC	1,439
5	5271–6755	TTTCTCGGGAGTAATAAAGAAGACGGTT	ACGCGTTCTCGTCTTTACTACTGG	1,485
6	6,614–8,096	AGTAGCTCCTCGTCAATATC	CAAACCCGTCGTATTTAGATAA	1,483
7	7995–9516	CGGTAAATTTAACGCTCAGA	CGAAGAACTCATAGAAAGCAT	1,522
8	9421–10853	TGTCTAAGGTTGGCTTGAAT	CTCACCGATTGTTCCTACTC	1,433
9	10747–12154	CTATCCATGAGTACCGTGTT	TTGAGTCTTTGCCATTTGAG	1,408
10	12047–13494	CAACAACTGTCGGCTATATG	AGCTAGGATTCTTCATCTGAG	1,448
11	13344–14846	CTGAGAAACGCTTTGAGAGA	CTTGACGCCTATATCCGATT	1,503
12	14703–16222	AGTATAAGTGGTACGATATAGCC	TGAGATTGCCTCTCTGAAAG	1,520
13	16101–17618	CCGCCTTCCTAGAATTAGAA	CATCCGTCTGAATAGGGTAA	1,518
14	17514–19022	TTTGATCTGAGGAATAAGTTTAGG	CGTATATGAGCCAGGAAGTA	1,509
15	18914–20484	GTATAGGTCTCAGTTACTAGGTA	TCTCTTGAGTAGTTGATCGTAT	1,571
16	20316–21820	CTCTTCCGCCATCATCAATA	CGATCTCATCCCGAATTTAGA	1,505
17	21719–23247	ACTCGGCCCTATGATAAATC	GTCTCCGTTCGTATCAGATA	1,529
18	23137–24671	CGACGGTGAAGAAAACGCAGA	CATGGTTCAGGAAGAAATAGG	1,535
19	24575–26097	CCTTCGGATATGATCCCTTTA	CTCTGAGCATGGTGAACTATA	1,523
20	25977–27486	GAGAGTATTACCAGACACCT	ACACCTATTCCACCATTAGT	1,510
21	27415–28932	AGGAATGGTAGATGGTAGATT	CCACTCCTAATCCTTCTTGA	1,518
22	28851–30309	GGAGGAATAGAATCATCAGAAG	GAACGATATGGAAGAGATTAGAG	1,459
23	30258–31729	TAACATCTCCTCATCCTCATC	TGAAATCAATTCCAAGTATTCCA	1,472
24	31578–33084	GGGAAGAAATAAACGGTATCG	GATTGCTCGGAGATGATGAT	1,507
25	32972–34479	GTGCTACCGTTGTAAACATTA	CGAATACATTACCAGTAACATAGA	1,508
26	34378–35877	TCCAAATTGTATCCACATAAAGT	CCACTATCATTACCACCACTA	1,500
27	35765–37219	CATTGAAGCTGTACTGGTGGAA	GAAGGCATTGCTAAGAGAAGGAT	1,455
28	37104–38660	CCATTGAAAGCATTTGTTGTTA	TGAATATGAAGAAGAGTGTGTTT	1,557
29	38499–39507	ATGCTCCATTGACTTATTCTG	CTCATGGTGTCACTATCTATTG	1,009
30	39340–40807	AAGGACCGATCTATAAACAGTA	AGAGCCTATTGGAAGATATGT	1,468
31	40638–42058	AGATGAAAGATAGGCAAGACA	ATTGGTTGAATGGCAGAGTA	1,421
32	41991–43181	CGCATAGAGTTCATTTCACAT	TCAGAGAATCATAATCGGAGAT	1,191
33	42506–43480	CAGCCAATGGAAAATCAGAG	CATCATCATATATAAAATAACCACAAAAC	975

**Table 3:** Orientations, locations, and amino acid (aa) sizes of predicted protein-coding regions in the CH-FJZZ-202201 and P29 genomes.

Gene	Strand^a^	GoAdV (P29)	No. of aa	GoAdV (CH-FJZZ-202201)	No. of aa
ORF1	R	795–1,229	144	799–1,233	144
ORF51	R	407–724	84	407–538; 602–724	84
ORF52	R	1,226–1,831	201	1230–1,835	201
ORF1C	R	1,791–1,931	46	1795–1,935	46
ORF2	R	2,012–2,854	280	2016–2,858	280
ORF14	L	2,866–3,591; 14,110–14,115	243	2871–3,596; 14121–14126	243
ORF12	L	3,603–4,514; 14,110–14,115	305	3608–4,519; 14121–14126	305
IVa2	L	4,471–5,667	398	4476–5672	398
pol	L	5,633–9,397	1,254	5638–9405	1,255
pTP	L	9,400–11,205; 14,110–14,115	603	9408–11216; 14121–14126	604
52K	R	11,232–12,383	383	11243–12394	383
pllIa	R	12,367–14,094	575	12378–14105	575
Ⅲ	R	14,131–15,717	528	14141–15727	528
pVII	R	15,722–15,976	84	15732–15986	84
pX	R	16,023–16,580	185	16033–16590	185
pVI	R	16,649–17,329	226	16663–17343	226
Hexon	R	17,371–20,172	933	17385–20186	933
Protease	R	20,186–20,806	206	20200–20820	206
DBP	L	20,877–22,273; 22,392–22,602	535	20889–22,279; 22398–22608	533
100K	R	22,660–25,602	980	22664–25609	981
22K	R	25,205–25,798	197	25197–25805	202
33K	R	25,205–25,640; 25,827–26,074	227	25197–25,647; 25,834–26081	232
pVIII	R	26,096–26,821	241	26103–26,828	241
U	L	<26,828–27,073	—	<26,835−27080	—
Fiber-1	R	27,097–28,377	426	27104–28384	426
Fiber-2	R	28,367–29,749	460	28374–29777	467
ORF22	L	29,741–30,352	203	29781–30389	202
ORF20A	L	30,355–31,131; 32,172–32,192	265	30392–31,165; 32206–32226	264
ORF20	L	31,116–32,069; 32,172–32,192	324	31150–32,103; 32206–32226	324
ORF56	L	32,263–32,745; 39,233–39,277	175	32297–32791 39279–39323	179
ORF55	L	32,697–33,101; 39,233–39,277	149	32,743–33,141; 39,279–39,323	147
ORF19	L	33,092–35,005; 39,233–39,277	652	33132–35,042; 39279–39,323	651
ORF54	L	34,999–35,388; 39,233–39,277	144	35036–35,425; 39279–39323	144
ORF19B	L	35,395–39,162; 39,233–39,277	1,270	35432–39,208; 39279–39323	1,273
ORF53	L	40,451–41,080	209	40497–41126	209

^a^R-rightward-transcribed strand and L-leftward-transcribed strand.

## Data Availability

All data are available from the authors upon reasonable request.

## Abbreviations

AvAstV: Avian astrovirus
DAdV: Duck adenovirus
EDSV: Egg drop syndrome virus
FaAdV: Falcon adenovirus
FAdV: Fowl adenovirus
GEFs: Goose embryo fibroblasts
GHPV: Goose hemorrhagic polyomavirus
GoAdV: Goose adenovirus
GPV: Goose parvovirus
HPS: Hydropericardium syndrome
IBH: Inclusion body hepatitis
PBS: Phosphate-buffered saline
PiAdV: Pigeon adenovirus
PsAdV: Psittacine adenovirus
REOV: Reovirus
RT-PCR: Reverse transcription-polymerase chain reaction
TAdV: Turkey adenovirus
TEM: Transmission electronic microscope
TMUV: Tembusu virus.

## Additional Points

*Highlights*. GoAdV-4 was first isolated from diseased Lion head gooses in China. The virions of GoAdV-4 were first studied by electron microscopy. The complete genome of the second GoAdV-4 strain was obtained worldwide.
